# Examination of Parental Effect on the Progeny Diapause by Reciprocal Cross Test in the Cabbage Beetle, *Colaphellus bowringi*

**DOI:** 10.1673/031.011.14501

**Published:** 2011-11-02

**Authors:** Cong-Hui Ma, Nan Ding, Xiao-Ping Wang, Chao-Liang Lei

**Affiliations:** Hubei Insect Resources Utilization and Sustainable Pest Management Key Laboratory, College of Plant Science and Technology, Huazhong Agricultural University, Wuhan 430070, China

**Keywords:** diapause induction, mass selection against diapause, photoperiod, temperature

## Abstract

The cabbage beetle, *Colaphellus bowringi* Baly (Coleoptera: Chrysomelidae), a serious pest of crucifers in China, undergoes summer or winter diapause in the soil as an adult. In the present study, the incidence of diapause were measured in reciprocal crosses between a high—diapause strain (HD strain) and a laboratory—selected nondiapausing strain (ND strain) under different photoperiods and temperatures, to explore parental influences on the progeny diapause. Sensitivity to photoperiod in the selected nondiapausing strain was nearly eliminated at 25 °C, whereas sensitivity to temperature of the selected nondiapausing strain was retained under continuous darkness at 20 and 22 °C. Reciprocal crosses between the HD strain and the ND strain showed that the incidence of diapause in the progeny was always intermediate to that of the parents under different photoperiods and temperatures, suggesting that diapause induction was determined by both female and male parents. There was a significant effect of temperature; temperature interacted with reciprocal cross on diapause induction, whereas no significant effect of reciprocal cross was demonstrated. The incidence of diapause in ♀_ND_ × ♂_HD_ was the same as in ♀_HD_ × ♂_ND_ under continuous darkness at 18 °C (100%) and 26 °C (0%), but the former was higher than that in ♀_HD_ × ♂_ND_ under continuous darkness at 22 °C, suggesting that female parent does not exhibit strong influence on the diapause response to temperature. There was a significant effect of photoperiod and reciprocal cross on diapause induction, whereas no significant interactive effect on diapause induction was demonstrated. Incidence of diapause in ♀_HD_ × ♂_ND_ was always higher than in ♀_ND_ × ♂_HD_ at 25 °C and 12:12 L:D, 14:10 L:D, and 16:8 L:D, suggesting a strong maternal influence on the diapause response to photoperiod, though a significant difference was observed only at 14:10 L:D. Our results support the idea that diapause induction is determined by both female and male parents. However, results also indicated that a strong maternal influence on diapause was exhibited only in response to photoperiod.

## Introduction

Many insects enter a diapause that allows them to survive harsh seasonal conditions (Denlinger 2002). Photoperiod and temperature, interacting with genetic components, are the main environmental cues known to be involved in the regulation of diapause (Masaki 1980; Saunders 2002). Genetic analysis of diapause or non—diapause traits is an available approach to understand the mechanism of seasonal adaptation of insects. Successful attempts to select for non—diapause or diapause traits, and reciprocal crosses under laboratory conditions have been reported in a variety of insect species (Tauber et al. 1986; Danks 1987; Socha and Hodkova 1994; Yang et al. 2007; Han and Denlinger 2009).

The cabbage beetle, *Colaphellus bowringi* Baly (Coleoptera: Chrysomelidae), is a serious pest of cruciferous vegetables in mountainous areas of China. The beetles enter summer and winter diapause as adults in the soil and show a great difference in diapause duration, ranging from several months to more than three years (Xue et al. 2002a). Temperature (or thermoperiod), photoperiod, and host plants are involved in diapause induction in this particular species (Xue et al. 2002b; Wang et al. 2006a, 2007). Generally, summer diapause in the cabbage beetle is induced by low temperature or by a mild temperature combined with long day length, while winter diapause is induced by low temperature regardless of photoperiod (Xue et al. 2002b). All adults enter diapause when the temperature is lower than or equal to 20 °C, regardless of the photoperiod. Its photoperiodic response is highly dependent upon temperature. The diapause—averting influences of short days are expressed only at temperatures above 20 °C (Xue et al. 2002b; Wang et al. 2004).

Previous studies with the cabbage beetle suggested that diapause is determined by both female and male parents, and there is a stronger maternal influence on the diapause compared to males (Yang et al. 2007; Lai et al. 2008). However, non-diapause trait selection (Yang et al. 2007) and reciprocal cross tests were performed under the most effective diapause-averting condition (25 °C and 12:12 L:D) (Yang et al. 2007; Lai et al. 2008). Moreover, the post—diapause adults were sexed and assigned directly in previous reciprocal crosses, and difference of parental physiological age and environmental experience among adult individuals was not ruled out in these two studies (Yang et al. 2007; Lai et al. 2008). Therefore, selection against diapause under strong pressure, elimination of difference among parental adult individuals before reciprocal crosses, and reciprocal cross tests performed under different conditions are necessary to verify previous results.

In the present study, we measured the incidence of diapause in reciprocal crosses between a high—diapause strain and a laboratory—selected nondiapausing strain under different photoperiods and temperatures to explore parental influences on sensitivity to diapause induction in progeny of *C. bowringi.*

## Materials and Methods

### High diapause strain

Post—diapause adults that entered diapause in late November 2008 were collected from the field in Xiushui County (29° 1′ N, 114° 4′ E), Jiangxi Province, China, and emerged from the soil in early October 2009. They were moved to plastic containers (7.5 cm × 7.5 cm × 6 cm) and mass reared for mating and oviposition. Eggs laid on the first three days were collected, and larvae were reared in plastic containers under diapause-averting short—day conditions (12:12 L:D at 25 °C). During the pupal stage, sex was determined according to Wang et al. (2006b). Female and male pupae or adults were then reared separately in plastic containers (7.5 cm × 7.5 cm × 6 cm) lined with layers of soil and fresh radish leaves, so that we could ensure that beetles used in experiments were virgins. The nondiapausing females and males (HD strain) used in this experiment were all seven—day— old virgins.

### Establishment of a nondiapausing strain

The beetles used for selection of non— diapause originated from a natural diapause population from Xiushui County (29° 1′N, 114° 4′ E), Jiangxi Province, China, collected in late November 2006. When the post—diapause adults were reared at a weak diapause inducing condition (22 °C and 13:11 L:D), some individuals developed without diapause. Nondiapausing females and males were collected and maintained under the same conditions for three generations. Nondiapausing individuals were then reared under the most effective diapause inducing condition (22 °C and 16:8 L:D) for continuous reproduction. When this selection was performed in the 22^nd^ generation, only 4.95% of individuals entered diapause.. During the pupal stage of the 23^rd^ generation, sex was determined according to Wang et al. (2006b). Then, female and male pupae or adults were reared separately. Nondiapausing females and males (ND strain) were also seven—day—old virgins.

### Comparison of incidence of diapause between the HD strain and ND strain

Eggs produced by the post-diapause adults (HD strain) and eggs of non-diapause adults of the 22^nd^ generation (ND strain) were collected at the same time. Then, the cabbage beetles were reared under conditions of 25 °C and 12:12 L:D or 16:8 L:D, and under continuous darkness at 20 or 22 °C for comparing the incidence of diapause.

### Crosses

Pure strains and reciprocal parental crosses were made as follows: (a) ♀_HD_ × ♂_HD_, (b) ♀_ND_ × ♂_ND_, (c) ♀_ND_ × ♂_ND_ and (d) ♀_ND_ × ♂_HD_. In the reciprocal cross experiments, we introduced a virgin HD female or ND female to a Petri dish and then added a virgin ND male or HD male. At least 50 pairs were obtained in each cross. The progeny of these crosses were reared under different photoperiods and temperatures.

### Insect rearing and conditions

Adults and larvae were reared on radish *Raphanus sativus* L. var. *longipinnatus* (Brassicales: Brassicaceae) in this study. To eliminate the influence of leaf aging during the experiment, host plants were sown at five—day intervals and mature leaves were collected daily for experiments. Eggs were collected for mass rearing. Just after hatching, at least 100 newly—hatched larvae were transferred to one transparent plastic container containing a layer of soil and radish leaves and then were placed at constant conditions until diapause was determined according to the criteria described below. Fresh mature leaves were provided daily. Three replications were performed for each treatment or generation. All experiments were conducted in an SPX—250IC illuminated incubator (Boxun Medical Instruments). In the illuminated incubators, light intensity during the photophase was approximately 2.0 W/m^2^, the temperature variation was approximately ± 1°C, and RH was approximately 70 ± 10%. Methods for scotophase control and the replenishments of rations were according to Xue et al. (2002b) and Wang et al. (2004).

### Diapause determination

All diapausing adults exhibit digging behavior and burrow into the soil after 4–6 days of feeding at 25 °C, 7–9 days at 20 °C, and 14–16 days at 15 °C regardless of photoperiod (Xue et al. 2002b). Therefore, diapause determinations were made after feeding for six days at 25 and 26 °C, nine days at 22 °C, and fourteen days at 18 °C.

### Statistical analysis

SPSS 11.5 (IBM, www.ibm.com) was used to perform analysis of variance (GLM: type III sum of squares) to determine the effects of temperature/photoperiod, cross (♀_HD_ × ♂_ND_ and ♀_ND_ × ♂_HD_), and their interaction on the incidence of diapause (arcsin—square root transformed). Meanwhile, the incidence of diapause (arcsin-square root transformed) at a given photoperiod or temperature was analyzed by ANOVA, and means were compared by Tukey's honestly significant difference test at α = 0.05.

**Table 1.**
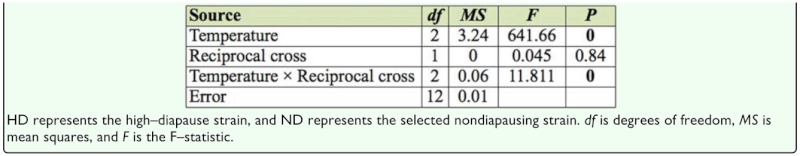
Analysis of variance (type III sum of squares) for the effect of temperature (18, 22 and 26 °C) and reciprocal cross (♀_HD_ ' ♂_ND_ and ♀_ND_ ' ♂_HD_) on the incidence of diapause in the cabbage beetle under continuous darkness.

**Table 2.**
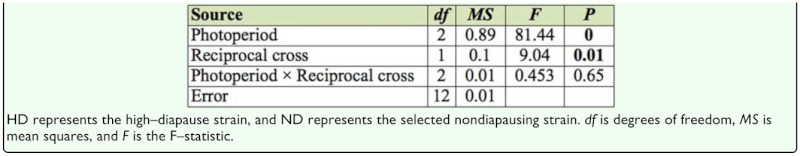
Analysis of variance (type III sum of squares) for the effect of photoperiod (12:12 L:D, 14:10 L:D and 16:8 L:D) and reciprocal cross (♀_HD_ ' ♂_ND_ and ♀_ND_ ' ♂_HD_) on the incidence of diapause in the cabbage beetle at 25 °C.

## Results

**Figure 1.**
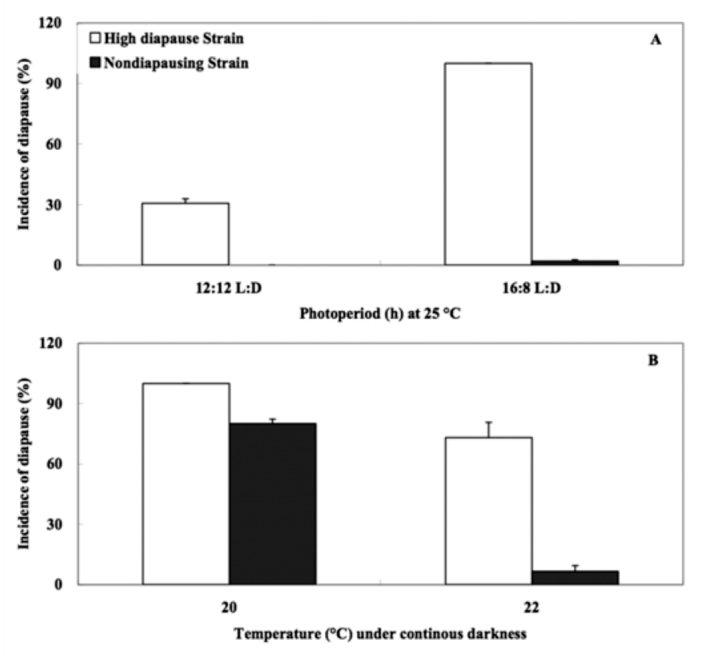
Comparison of incidence of diapause between the selected nondiapausing strain and high-diapause strain of the cabbage beetle (A) at 25 °C and (B) under continuous darkness. Three replications were performed for each treatment and error bars indicated SD. N ranged from 163 to 203 for each treatment. High quality figures are available online.

The incidence of diapause in the HD strain and ND strain of the cabbage beetle were compared before reciprocal crosses. Incidence of diapause in the HD strain were significantly higher than those of the ND strain under all testing conditions (*t*-test, *p* < 0.05). At 25 °C, 100 and 30.6% of HD adults entered diapause under 16:8 L:D and 12:12 L:D, respectively, whereas only 2% of ND adults entered diapause under 16:8 L:D. No adults entered diapause at 12:12 L:D in the ND strain (Figure 1A), showing that the ND strain was insensitive to photoperiod. However, under continuous darkness, incidence of diapause were 100% at 20 °C and 73.1% at 22 °C in the HD strain, and 80.0% at 20 °C and 6.6% at 22 °C in the ND strain, respectively (Figure 1B), showing that sensitivity to temperature is retained in the ND strain.

**Figure 2.**
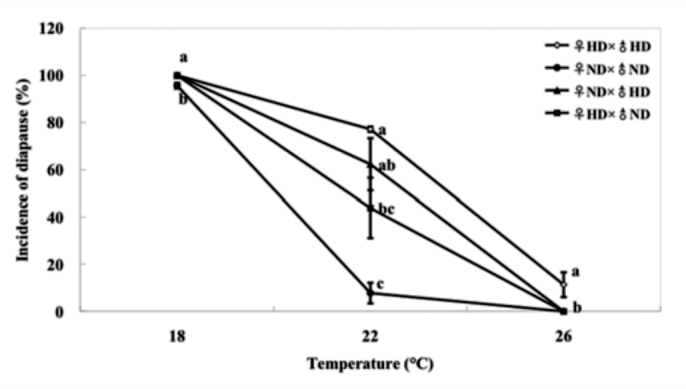
Incidence of diapause of crosses between a selected nondiapausing strain (ND strain) and a high—diapause strain (HD strain) of the cabbage beetle at different temperatures combined with continuous darkness. Three replications were performed for each treatment and error bars indicate the SD. N ranged from 119 to 274 for each treatment. Bars with the same letter at same temperature are not significantly different by Tukey's honestly significant difference test (*p* > 0.05). High quality figures are available online.

There was a significant effect of temperature; temperature interacted with reciprocal cross on diapause induction, whereas no significant effect of reciprocal cross was demonstrated (Table 1). Temperature was the major determinant of diapause induction in the cabbage beetle, and the incidence of diapause was highest at 18 °C (95.6–100%), followed by 22 °C (7.6–77.1%), and 26 °C (0–11.4%) (Figure 2). Incidence of diapause of different crosses differed significantly at 18 °C (*F*3,11 = 155.528, *p* < 0.01), 22 °C (*F*3,11 = 38.235, *p* < 0.01), and 26 °C (*F*3,11 = 43.962, *p* < 0.01). However, incidence of diapause of two reciprocal crosses did not differ significantly at the same temperature. Incidence of diapause in ♀_ND_ × ♂_HD_ was the same as in ♀_HD_ × ♂_ND_ at 18 °C (100%) and at 26 °C (0%). The incidence of diapause of reciprocal crosses were intermediate between either parental strain, and the incidence of diapause in ♀_ND_ × ♂_HD_ was higher than that in ♀_HD_ × ♂_ND_ at 22 °C without the participation of illumination, also demonstrating that there was a temperature × reciprocal cross interaction on the incidence of diapause (Figure 2). This suggests that diapause was determined by both parents, but no stronger influence of either parent was demonstrated in response to temperature.

**Figure 3.**
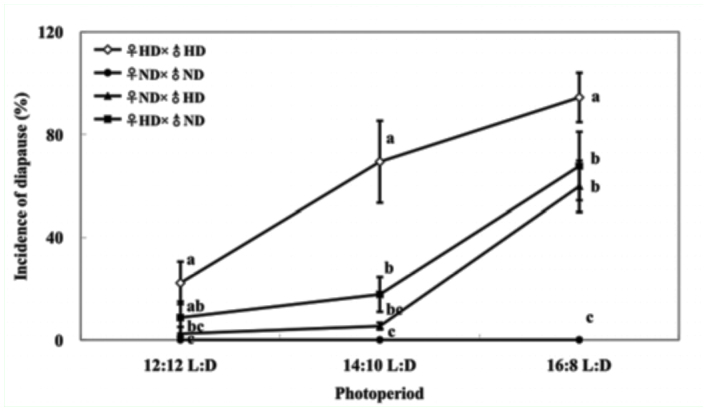
Incidence of diapause of crosses between a selected nondiapausing strain (ND strain) and a high—diapause strain (HD strain) of the cabbage beetle at 25 °C and different photoperiods. Three replications were performed for each treatment and error bars indicate the SD. N ranged from 136 to 243 for each treatment. Bars with the same letter under same photoperiod are not significantly different by Tukey's honestly significant difference test (*p* > 0.05). High quality figures are available online.

There was a significant effect of photoperiod and reciprocal cross on diapause induction, whereas no significant interactive effects on diapause induction were demonstrated (Table 2). Incidence of diapause of different crosses differed significantly at 12:12 L:D (*F*3,11 = 14.244, *p* < 0.01), 14:10 L:D (*F*3,11 = 55.752, *p* < 0.01), and 16:8 L:D (*F*3,11 = 48.158, *p* < 0.01) (Figure 3). The incidence of diapause of reciprocal crosses was intermediated between the HD strain and the ND strain. The incidence of diapause in ♀_HD_ × (♂_ND_ was always higher than that in ♀_ND_ × ♂_HD_, but there were no significant differences in any tested photoperiods (Figure 3). These results suggested that diapause was determined by both parents, and that female parents showed a stronger effect in response to photoperiod.

## Discussion

To understand the genetic and regulating mechanism of diapause, selection for a nondiapause or diapause strain under laboratory conditions has been reported in a variety of insect species, and most frequently, selection to eliminate diapause has been made (Tauber et al. 1986; Danks 1987; Socha and Hodkova 1994). Diapause proneness can be rapidly altered by artificial selection, whereas it has rarely been eliminated completely over a modest number of generations in most species (Tauber et al. 1986; Danks 1987). In the cabbage beetle, the diapause response to artificial selection was also rapidly weakened and was eliminated completely, irrespective of photoperiods, except at 16:8 L:D in the tenth generation at 25 °C (Xue et al. 2002b). A non-diapause strain was also obtained after successive selection for 37 generations at 25 °C and 12:12 L:D (Yang et al. 2007). However, the selection pressure was not sufficient for selection of non-diapause because the conditions of 25 °C and 12:12 L:D were most effective for averting diapause (Xue et al. 2002b; Wang et al. 2004). In the present study, non-diapause was selected at 22 °C and 16:8 L:D, in which all adults entered diapause in the wild strain (Xue et al. 2002b). Our results further showed that sensitivity to photoperiod in the cabbage beetle could be eliminated by artificial selection, whereas sensitivity to temperature was retained but somewhat reduced (Figure 1). In addition, we failed to obtain a low diapause strain at 18 °C since the temperature decreases progressively generation—by— generation. Only 3.6% of the reproductive individuals were obtained after eight generations of selection. However, the reproductive adults also diapaused after a period of egg—laying at 18 °C (unpublished data). In fact, the adult stage was sensitive to low temperature but not to photoperiod in diapause induction (Xue et al. 2002b), and nondiapausing adults could be induced to enter diapause after a period of egg—laying in response to low temperature (Wang et al. 2005). These results were in agreement with the data of Xue et al. (2002b), suggesting that photoperiodic and temperature controls of diapause induction might have different genetic bases in the cabbage beetle.

In insects, both the expression and the interaction with environmental factors of the various components of diapause are under genetic control. Evidence about the genetics of diapause has been obtained chiefly by crossing different geographic or laboratory strains with different diapause responses, and genetic studies related to diapause have shown various modes of inheritance in different species (Tauber et al. 1986; Danks 1987; Takeda 1998; Han and Denlinger 2009). Patterns of inheritance consistent with polygenic systems have been reported in the majority of species that have been investigated, and diapause characteristics of hybrids often are intermediate between those of the parents (Danks 1987). Single reciprocal crosses between the selected strain and a high-diapause strain in the cabbage beetle showed that the hybrids had an intermediate response, similar to many other insect species (Simis 1983; Wipking and Kurtz 2000; Tan et al. 2008), indicating that diapause induction was influenced by both parents in this species. These results are in accord with those of previous studies obtained by crossing different geographic and laboratory strains with different diapause responses (Yang et al. 2007; Lai et al. 2008).

Previous studies reported a stronger maternal influence on progeny diapause than from the male parent in the cabbage beetle (Yang et al. 2007; Lai et al. 2008). Our results indicated that the incidence of diapause in two reciprocal crosses were intermediate between the parental strains. There was a stronger maternal influence on progeny diapause response to photoperiod than from the male parent in the cabbage beetle, but no significant difference was found. However, this stronger maternal influence on progeny diapause was not exhibited in response to temperature. We presumed that the experiences of parents were the causes of this contradiction, because postdiapause adults were used in previous studies directly (Yang et al. 2007; Lai et al. 2008). In order to rule out the difference of parental physiological age and environmental experience among adult individuals in the present study, cabbage beetles were reared under the same conditions for one generation before reciprocal cross tests. In previous studies (Yang et al. 2007; Lai et al. 2008), reciprocal cross tests were performed only under the most effective diapause-averting condition (25 °C and 12:12 L:D), whereas different photoperiods and temperatures were used in the present study.

Therefore, we conclude that diapause is determined by both female and male parents, but stronger maternal influence on the diapause was exhibited only in response to photoperiod in the cabbage beetle.

## Abbreviations

**HD strain**,: high-diapause strain;
**ND strain**,: Nondiapausing strain
